# Deletion of myeloid IRS2 enhances adipose tissue sympathetic nerve function and limits obesity

**DOI:** 10.1016/j.molmet.2018.11.010

**Published:** 2018-11-28

**Authors:** Marie-Therese Rached, Steven J. Millership, Silvia M.A. Pedroni, Agharul I. Choudhury, Ana S.H. Costa, Darran G. Hardy, Justyna A. Glegola, Elaine E. Irvine, Colin Selman, Megan C. Woodberry, Vijay K. Yadav, Sanjay Khadayate, Antonio Vidal-Puig, Samuel Virtue, Christian Frezza, Dominic J. Withers

**Affiliations:** 1 MRC London Institute of Medical Sciences, Du Cane Road, London, W12 0NN, UK; 2 Institute of Clinical Sciences, Faculty of Medicine, Imperial College London, Du Cane Road, London, W12 0NN, UK; 3 MRC Cancer Unit, University of Cambridge, Box 197, Cambridge Biomedical Campus, Cambridge, CB2 0XZ, UK; 4 Institute of Biodiversity, Animal Health and Comparative Medicine, College of Medical, Veterinary and Life Sciences, University of Glasgow, Glasgow, G12 8QQ, UK; 5 Wellcome Trust Sanger Institute, Hinxton, Cambridge, CB10 1SA, UK; 6 Department of Genetics and Development, Columbia University, New York, 10032, USA; 7 University of Cambridge Metabolic Research Laboratories, Wellcome Trust-MRC Institute of Metabolic Science, Cambridge, CB2 0QQ, UK

**Keywords:** Macrophage, *Irs2*, Obesity, Inflammation, BAT, Sympathetic neurons, WAT, white adipose tissue, BAT, brown adipose tissue, CAM, classically activated macrophages, AAM, alternatively activated macrophages, HFD, high fat diet, EE, energy expenditure, HOMA-IR, homeostatic model assessment-insulin resistance, BMDM, bone marrow-derived macrophages, LPS, lipopolysaccharide, ATM, adipose tissue macrophages, SAM, sympathetic-associated macrophages, CLAMS, comprehensive lab animal monitoring system, SVF, stromal vascular fraction

## Abstract

**Objective:**

Sympathetic nervous system and immune cell interactions play key roles in the regulation of metabolism. For example, recent convergent studies have shown that macrophages regulate obesity through brown adipose tissue (BAT) activation and beiging of white adipose tissue (WAT) via effects upon local catecholamine availability. However, these studies have raised issues about the underlying mechanisms involved including questions regarding the production of catecholamines by macrophages, the role of macrophage polarization state and the underlying intracellular signaling pathways in macrophages that might mediate these effects.

**Methods:**

To address such issues we generated mice lacking *Irs2*, which mediates the effects of insulin and interleukin 4, specifically in *LyzM* expressing cells (*Irs2*^*LyzM*−/−^ mice).

**Results:**

These animals displayed obesity resistance and preservation of glucose homeostasis on high fat diet feeding due to increased energy expenditure via enhanced BAT activity and WAT beiging. Macrophages per se did not produce catecholamines but *Irs2*^*LyzM*−/−^ mice displayed increased sympathetic nerve density and catecholamine availability in adipose tissue. *Irs2*-deficient macrophages displayed an anti-inflammatory transcriptional profile and alterations in genes involved in scavenging catecholamines and supporting increased sympathetic innervation.

**Conclusions:**

Our studies identify a critical macrophage signaling pathway involved in the regulation of adipose tissue sympathetic nerve function that, in turn, mediates key neuroimmune effects upon systemic metabolism. The insights gained may open therapeutic opportunities for the treatment of obesity.

## Highlights

• *Irs2* deletion in macrophages improves metabolic health and BAT activity in mice.
• *Irs2*-deficient BMDM are protected from M1 polarisation by LPS.
• BAT norepinephrine levels and TH density mirror increases in BAT activity.
• Cross-talk between macrophages and BAT sympathetic neurons underpin these findings.
• Macrophage *Irs2* modulates energy homeostasis.

## 1 Introduction

Obesity and its associated co-morbidities such as type 2 diabetes are major threats to health worldwide with over 2.1 billion people currently being classified as overweight or obese [1], [2], [3]. The pathophysiology of obesity is increasingly thought to involve cross-talk between the immune and metabolic regulatory systems [4], [5], [6], [7], [8], [9]. Over-nutrition, leading to obesity, is associated with an altered adipose tissue immune profile and inflammation [4], [7], [8], [9]. This in turn leads to a systemic pro-inflammatory state causing insulin resistance, a primary factor in the pathophysiology of type 2 diabetes [4], [6], [7], [8], [9]. Macrophages sit at the core of obesity-associated inflammation [6], [9]. While now recognized as an oversimplification, two basic polarization states of macrophages have been defined: classically activated (M1) macrophages (CAM) have a pro-inflammatory phenotype while alternatively activated (M2) macrophages (AAM) have an anti-inflammatory phenotype [10], [11]. The distinct physiological roles of these macrophage phenotypes are reflected in the pathogenesis of obesity which is associated with increased adipose tissue M1 macrophage infiltration and release of inflammatory cytokines leading to antagonism of insulin signaling [7], [8], [12]. In contrast, AAM can mitigate against insulin resistance with improved insulin sensitivity associated with increased numbers of AAM in adipose tissue [7], [8], [9]. Macrophage polarization status is under complex regulation with interferon gamma, for example, promoting the generation of CAM/M1 macrophages while interleukins (IL) IL4/IL13 promote the AAM/M2 phenotype [13], [14]. IL4/IL13 may play a role in metabolic homeostasis [13]. For example, maintenance of AAMs in white adipose tissue (WAT) depends on IL4/13 signaling to STAT6 [13], [15] and this pathway also programs macrophage metabolism via PPARG, PPARD, and PGC1B away from the anaerobic metabolic profile seen in CAM [13], [16]. IL4 signaling in macrophages has also been proposed to regulate BAT thermogenesis as acute cold stress causes AAM themselves to produce catecholamines in an IL4-dependent manner, leading to BAT browning and the biogenesis of beige fat in WAT depots [17]. Consistent with these observations, IL4 administration to mice provided protection from obesity-associated insulin resistance [17]. However, these findings have been called into question by a recent study that failed to confirm that AAM synthesize catecholamines or regulate adaptive thermogenesis and did not demonstrate beneficial metabolic effects of IL4 administration to mice [18]. In contrast, it has been demonstrated that macrophages may directly regulate sympathetic nervous system function in adipose tissue including the regulation of browning [19], [20] but the potential role of IL4 in this process is unclear.

In macrophages, IL4 signals via either the type I receptor consisting of IL4RA and IL4RG subunits or the type II receptor composed of IL4RA and IL13RA1 subunits [13], [21], [22], [23]. Type I and type II receptors recruit JAK/STAT signaling with the shared IL4RA chain associating with JAK1 leading to STAT6 phosphorylation [13], [21], [22], [23]. In addition, IL4RA activates insulin receptor substrate (IRS) 2, which is also the major target of insulin and IGF1 receptors in hematopoietic cells particularly of the myeloid lineage [24], [25], [26]. When tyrosine phosphorylated, IRS2 recruits the regulatory subunits of PI3K, GRB2, and other SH2 domain containing molecules to activate a number of downstream signaling pathways [27], [28]. However, despite its potentially critical role in both IL4 and insulin/IGF1 receptor signaling, the precise in vivo function of IRS2 in macrophage biology is less clear. Defining its role is complicated by the observations that while IL4 signaling is primarily anti-inflammatory, insulin action upon macrophages may be pro-inflammatory [21], [29], [30]. Thus, IRS2 signaling may mediate divergent biological outputs but to date the precise balance of such effects in metabolic physiology is unknown. For example, myeloid-restricted deletion of the insulin receptor in mice leads to improved whole animal insulin sensitivity under conditions of high fat feeding due to reduced migration of macrophages into white adipose tissue [31]. Mice with global deletion of *Irs2* develop progressive diabetes due to a combination of systemic insulin resistance, hypothalamic obesity and pancreatic beta cell failure which together have precluded detailed analysis of the specific role of macrophage IRS2 signaling in physiology [32], [33], [34]. However, a study using transplanted bone marrow from these animals demonstrated that insulin signaling via IRS2 in macrophages contributed to pro-inflammatory signals involved in the development of atherosclerosis [35]. Consistent with this concept, in vitro studies have suggested that IRS2 signaling in macrophages is required for IL4/IL4RA–mediated expression of genes characteristic of the AAM/M2 anti-inflammatory phenotype [21]. However, recent studies in *Irs2*-deficient macrophages also demonstrated alterations in gene expression towards an M2 phenotype with compensatory IRS1 signaling suggested to underlie this observation [29]. These findings also indicate that IRS2 signaling might act as a restraint to IL4-mediated regulation of M2 genes revealing unexpected complexities in the underlying signaling mechanisms.

Given the key potential role of IRS2 signaling as an intersection point between IL4 and insulin signaling in macrophage function and systemic metabolism we generated mice with myeloid-restricted deletion of *Irs2*. We found that macrophages from these animals showed a broad anti-inflammatory phenotype when exposed to inflammatory mediators but with a limited increase in the expression of markers of the AAM/M2 phenotype. Furthermore, these animals showed improved insulin sensitivity and a resistance to weight gain on a high fat diet associated with increased BAT function and increased WAT browning at ambient temperature. However, we were unable to find evidence that this phenotype was driven by intrinsic macrophage catecholamine production. In contrast, we find that macrophages associate with BAT sympathetic nerves and that deletion of *Irs2* in these cells increases sympathetic innervation and local norepinephrine levels in BAT. Together these studies reveal an important role for macrophage *Irs2* in systemic metabolism and reveal new details of the signaling mechanisms underlying the cross-talk between the immune and nervous system relevant to the pathogenesis of obesity and insulin resistance.

## 2 Methods

### 2.1 Animals

Experiments involving animals were designed and reported following the ARRIVE guidelines of animal experiment reporting [36]. Power calculations for number of mice for each experiment were based on reported or known effect sizes and variation, in order to maximize chances of meaningful results without the unnecessary use of experimental animals. Where possible, investigators were blinded to the genotype of both study animals and tissue/blood samples. BMDM studies were performed on 10-week old male mice.

Deletion of *Irs2* in myeloid lineages was carried out using *Cre*-*LoxP* technology using mice with a previously generated floxed allele of *Irs2* [37] crossed with mice expressing *Cre*-recombinase driven by the Lysozyme M promoter [38]. *Irs2* floxed mice were backcrossed to a C57BL/6J background whereas Lysozyme M *Cre* mice were on a mixed C57BL/6J:129S2/Sv genetic background. *Irs2* deletion and the presence or absence of the *Cre* recombinase were determined by PCR as described previously; the following PCR primers were used for amplification: *Irs2* flox forward: 5′-ACTTGAAGGAAGCCACAGTCG-3′, *Irs2* flox reverse: 5′-AGTCCACTTTCCTGACAAGC-3′, Lysozyme M *Cre* forward: 5′-CCCAGAAATGCCAGATTACG-3′, Lysozyme M *Cre* reverse: 5′- CTTGGGCTGCCAGAATTTCTC-3’ [37], [38].

Mice were maintained in a pathogen–free facility in individually ventilated cages under a controlled temperature between 21 °C and 23 °C and a regular 12 h light/dark cycle with ad libitum access to water and normal chow diet (11.5% energy from fat). The high fat diet (HFD, Research Diets, D12451) contained 45.0% energy from fat while the percentage energy from carbohydrates was less relative to the chow diet (35.0% versus 61.6% respectively). Mouse studies were performed in accordance to the United Kingdom Animals (Scientific Procedures) Act (1986) and approved by Imperial College London's Animal Welfare and Ethical Review Body.

### 2.2 Metabolic studies

Total fat and lean mass from live non-anesthetized mice were determined using an EchoMRI quantitative whole-body composition analyzer (Zinsser Analytic) and mice were subsequently weighed to determine percentage fat and lean mass per body weight. Energy expenditure was calculated from singly-housed mice in CLAMS cages (Columbus Instruments) at ambient temperature (22 °C) for 24 h following 24 h of acclimatization. Recordings were taken every 20 mins. During energy expenditure measurement, water and food were provided ad libitum. Assessment of food intake was performed in singly housed mice for 3 consecutive experimental days after a period of acclimatization [37].

High fat diet experiments were performed on 8-week-old control and *Irs2*^*LyzM*−/−^ mice, which were each divided into two weight-matched groups. Littermates of mixed genotypes were caged together and each group maintained on either chow or HFD. Fasted glucose measurements, glucose tolerance tests (GTTs) and insulin tolerance tests (ITTs) were performed as previously described [37], [39], [40]. GTTs on chow diet were performed by administering 2 g/kg of glucose per body weight intraperitoneally (i.p.) at 9am after an overnight fast whereas GTTs on HFD-fed mice were performed using glucose at a lower dose (1 g/kg bodyweight). For ITTs, food was removed from mice at 8am and the animals were injected i.p. with insulin (0.5 U/kg) at 2pm. Blood glucose was measured at the times indicated in the figures using a Contour glucometer (Bayer). Insulin sensitivity on HFD was assessed in 12 h-fasted mice by the homeostatic model assessment of insulin resistance (HOMA-IR) calculated as (I0 x G0)/405 where G0 is fasting blood glucose in mg/dl and I0 is fasting insulin in μIU/ml measured simultaneously [41].

For insulin and leptin measurements, serum from fasted mice was collected from tail vein bleeds into capillary tubes (Sarstedt) and spun for 15 min at 4 °C at 2000×*g*. The serum supernatant was removed into a new tube and stored at −80 °C. Hormones were measured by ELISA (Millipore) according to the manufacturer's instructions. BAT catecholamines were measured by 2-CAT (A-N) ELISA (Rocky Mountain Diagnostics) according to manufacturer's instructions. Briefly, BAT tissues were homogenized in homogenization buffer (1N HCl, 0.25M EDTA, 1M Na_2_S_2_O_5_), and homogenates were collected after centrifugation and stored at −80 °C prior to quantification. All samples were normalized to total tissue protein concentration.

### 2.3 Histology

Interscapular BAT, perigonadal WAT (pWAT), and inguinal WAT (iWAT) tissues were excised and fixed in 10% neutral buffered formalin for 24 h. Tissues were then dehydrated in increasing ethanol concentrations before being embedded in paraffin and serially sectioned (5 μm). Sections were deparaffinized with xylene and rehydrated before being stained with hematoxylin and eosin [42].

For immunohistochemistry, iWAT sections were stained with polyclonal anti-UCP1 antibody (Abcam) and macrophages in pWAT were immunostained with monoclonal rat anti-MAC2 antibody (Cedarlane Laboratories) as described previously [43], [44]. Detection was performed with an HRP-conjugated secondary antibody followed by chromogenic detection using DAB substrate. Brown and white adipose tissue images were taken at 20x magnification using a light microscope (Leica DM4000 B).

For immunofluorescence staining of tyrosine hydroxylase (TH) alone or both TH and CX3CR1 staining on BAT sections, BAT tissues were fixed with 4% paraformaldehyde (PFA) and then transferred to ethanol. Sections were antigen retrieved by boiling in 10 mM citrate buffer, pH 6, blocked with serum-free blocking agent (Invitrogen) and sequentially stained with rabbit anti-TH (Millipore) and rabbit polyclonal antibody to CX3CR1 (Abcam) [45]. Secondary antibodies, goat anti-rabbit AlexaFluor-488 and goat anti-rabbit AlexaFluor-568, were from Invitrogen.

For quantitative TH density analysis, a custom ImageJ macro was written. Briefly, tissue area was identified by thresholding low intensity autofluorescence signal after application of a median filter (r = 5), with the image subsequently converted to a binary mask. Further binary operations including morphological closing and dilation and fill holes, were used to accurately identify the boundaries of the total tissue area within the image. For subsequent analysis, pixels outside the tissue area segmented were set to zero. Identification of the tissue area without the inclusion of areas occupied by lipids was achieved by using a similar method as aforementioned but without the addition of the further binary operations. Fluorescence from TH immunostaining was analyzed according to these thresholds and binary operations made accordingly. Percentage of the fluorescent signal relative to the total tissue area excluding lipids is presented as TH density.

### 2.4 Magnetic isolation of CD11B positive fraction from the stromal vascular fraction of perigonadal WAT

Perigonadal white adipose tissue (pWAT) was collected from HFD-fed mice and digested in a Hank's Balanced Salt Solution containing BSA and type II collagenase (Sigma–Aldrich) at 37 °C, 100 rpm for 20–30 min. The floating adipocytes were separated from the stromal vascular fraction (SVF) by centrifugation. The CD11B fraction was extracted from the SVF pellet using CD11B microbeads (Miltenyi Biotec) conjugated to monoclonal rat anti-mouse/human antibody. The mixture of SVF/antibody was passed through a LS column (Miltenyi Biotec) placed in a magnetic field. CD11B positive fraction was eluted according to the manufacturer's instructions and then processed for RNA extraction using the RNeasy mini kit (Qiagen) [46].

### 2.5 Macrophage culture and treatment

Bone marrow-derived macrophages (BMDMs) were cultured as previously described [47]. Briefly, femurs and tibias were cut at the proximity of each joint and bone marrow flushed out with PBS. Cells were then collected, centrifuged at 500×*g*, and resuspended in macrophage complete medium (1:1 DMEM/F12, 10% FBS, 2 mM l-glutamine) consisting of 25% L929 conditioned-medium until fully differentiated (7–10 days). Macrophage activation was induced in differentiated BMDMs by stimulation with bacterial lipopolysaccharide (LPS) from Escherichia coli (100 ng/ml, Sigma–Aldrich) or IL4 (10 ng/ml, R&D Systems) for 24 h [10], [21], [48]. For short-term signaling studies, BMDMs were treated with IL4 (20 ng/ml) or insulin (10 nM, Novo Nordisk) for the times indicated and homogenized in lysis buffer (50 mM Tris–HCl pH 7.5,150 mM NaCl, 1% Triton-X100, 1 mM EDTA with Complete Mini protease inhibitors and PHOSstop phosphatase inhibitors (both Roche)). Phosphorylation of AKT was analyzed by western blotting from cleared lysates normalized for total protein by BCA method (Bio-Rad) using antibodies against serine 473 phosphorylated AKT and pan AKT (both Cell Signaling, 9271 and 2920, respectively). For expression of IRS1, lysates from brain tissue and BMDM, obtained as above, were blotted using a monoclonal IRS1 antibody (Upstate, clone 4.2.2). For palmitate treatment, BMDMs were incubated with 500 μM palmitate-BSA (Agilent technologies) for the times indicated, with BSA treatment as a control. BMDMs were then processed for RT-PCR studies to measure *Irs2* mRNA expression as described below.

### 2.6 Catecholamine measurement by LC/MS

500,000 cells were seeded in 6-well plates and treated for 24 h with either IL4 or LPS. Control (non-conditioned) and BMDM-conditioned medium was collected from each well, centrifuged at 16,000×*g* for 10 min at 4 °C and 50 μl of the supernatant were added to 750 μl of cold extraction solution (50% v/v methanol, 30% v/v acetonitrile, 20% v/v ddH_2_O). Cell culture medium extracts were kept under agitation at 4 °C for 15 min in a ThermoMixer (Eppendorf). Finally, the suspension was centrifuged for 10 min at 16,000×*g* at 4 °C, the supernatant transferred into autosampler vials and stored at −80 °C until further analysis. Liquid chromatography–mass spectrometry (LC–MS) analysis was performed on a Q Exactive mass spectrometer (Thermo Fisher Scientific) coupled to a Dionex UltiMate 3000 Rapid Separation LC system. The LC system was fitted with a Raptor FluoroPhenyl column (150 mm × 2.1 mm, 2.7 μm) with the corresponding guard column (both Restek). The mobile phase was composed of 0.2% formic acid in water and methanol. The flow rate was set at 400 μl min^−1^, with a gradient from 2% to 40% organic, and a total run time of 8 min. The mass spectrometer was operated in full MS and positive ionization mode. Samples were randomized, to avoid bias due to machine drift, and blinded to the operator. The acquired spectra were analyzed using XCalibur Qual Browser and XCalibur Quan Browser software (Thermo Scientific) by referencing to an internal library of compounds and synthetic standards run with the same batch as the samples.

### 2.7 Immunocytochemistry for IRS2 in BMDM

BMDM were plated on Matrigel-coated glass coverslips (VWR). Once adhered, cells were fixed with 100% methanol for 1 min at room temperature. For IRS2 staining, cells were permeabilized (PBS with 0.1% Triton X-100 and 1% BSA) for 5 min. After fixing and permeabilization, coverslips were washed (PBS) and blocked (0.5% fish skin gelatin in PBS) overnight. Coverslips were then incubated with rabbit polyclonal anti-IRS2 primary antibody (Upstate) for 3 h at room temperature. Cells were washed and further incubated with goat anti-rabbit AlexaFluor-488 secondary antibody (Invitrogen) for 30 min in the dark. Cover slips were washed with PBS to remove unbound secondary antibody and mounted with Vectashield® mounting medium with DAPI (Vector labs) on glass slides, immobilized with clear nail polish, and visualized using a Leica SP5 confocal microscope.

### 2.8 mRNA expression analysis and RNA sequencing

RNA was extracted from snap-frozen tissues and BMDM using TRIzol reagent (Sigma–Aldrich) followed by chloroform treatment, and isopropanol to precipitate RNA. Following centrifugation, the pellet was washed with 70% ethanol, dried and resuspended in RNase-free H_2_O. RNA quality and quantity were assessed using a ND-1000 spectrophotometer Nanodrop (Thermo Fisher Scientific) for real-time PCR (RT-PCR) purposes and a Bioanalyzer 2100 (Agilent) for mRNA sequencing purposes. RNA from the cell pellet collected from CD11B positive fractions was isolated using RNeasy spin columns (Qiagen).

Total RNA was treated with DNase I (Qiagen) and 1 μg was reverse transcribed at 42 °C with SuperScript II (Invitrogen). Quantitative PCR reactions were carried out in duplicate on 384-well clear optical plates using a Taqman ABI 7900HT fast real-time PCR machine equipped with SDS 2.3 software. A list of all probes is presented in Supplemental Table 1. Experimental Ct values were normalized to specific housekeeping genes. A relative quantification using the ΔCT method or an absolute quantification using the standard curve method were used as appropriate as indicated in figure legends. Hypoxanthine phosphoribosyltransferase (*Hprt*) was the housekeeping gene used to normalize Ct values in macrophages. *Hprt* Ct values were stable across BMDM and ATM samples of both genotypes and upon IL4 and LPS treatments. To quantify mRNA in adipose tissues, the housekeeping gene *Gapdh* (glyceraldehyde 3 phosphate dehydrogenase) was used.

For RNA sequencing analysis, the standard Illumina library preparation protocol was carried out on 2 μg of RNA and the library prepared using the TrueSeq kit (Illumina) according to manufacturer's protocol. Each sample was divided into 8 technical replicates and sequenced on individual lanes in an Illumina flow cell. The sequencing was performed using standard Illumina protocols at the Sanger institute. Raw reads from the sequenced RNAseq libraries were mapped with Tophat splice junction aligner [49], version 2.0.8 against Ensembl mouse genome reference sequence assembly (mm9) and transcript annotations. All parameters were set to default except inner distance between mate pairs (r = 130).

Gene-based read counts were then obtained using the HTSeq count module (version 0.5.4p3) [50]. This HTSeq counts module was run using parameters m = intersection-nonempty, stranded = no. Differential expression analysis was performed on the counts data using the DESeq2 Bioconductor package (version 1.16.1) [51]. This DESeq2 package uses negative binomial model to model read counts and then performs statistical tests for differential expression of genes. Raw P values were then adjusted for multiple testing with the Benjamini-Hochberg procedure. Genes with adjusted P value of 0.05 or less were termed as differentially expressed genes.

Geneset enrichment analysis was performed to elucidate biological pathways and processes regulated by the interaction between *Irs2*^*LyzM*−/−^ and control BMDM and with various treatments (IL4, LPS). The GSEA [52] preranked tool from GSEA version 2.2.4 was used for this purpose. Gene ontology enrichment analysis was also performed using the topGo Bioconductor package [53] separately for up and down-regulated genes in the comparisons analyzed. RNA sequencing data is available from GEO accession GSE 123180.

### 2.9 Statistics

Statistical analysis was carried out using Prism 7 software. Student's t test or ANOVA (or Mann–Whitney U or Kruskal–Wallis tests) were used where data was parametric or nonparametric, respectively. A P value less than 0.05 was considered significant where * represents P < 0.05, **p < 0.01 and ***p < 0.001. In all panels, data are represented as mean ± SEM unless otherwise stated. For energy expenditure (CLAMS) data on HFD, a general linear modeling (GLM) in SPSS was used with genotype as a fixed factor, body weight as covariate and energy expenditure as the dependent variable.

## 3 Results

### 3.1 Loss of *Irs2* in myeloid cells improves glucose homeostasis and promotes resistance to high fat diet induced metabolic dysfunction

To investigate the role of macrophage IRS2 signaling in metabolism, we conditionally deleted *Irs2* in myeloid cells in mice. To achieve this, we generated mice homozygous for a floxed allele of *Irs2* [37] and heterozygous for a transgene expressing *Cre* recombinase from the *LyzM* locus (*Irs2*^fl/fl^*LyzM*-*Cre*^tg/wt^ termed *Irs2*^*LyzM*−/−^ hereafter) [38]. A combination of pure WT, *Irs2*^fl/fl^ and *LyzM-Cre*^tg/wt^ which were phenotypically indistinguishable served as controls. We confirmed efficient deletion of *Irs2* in bone-marrow derived macrophages (BMDM) by analyzing mRNA expression levels in *Irs2*^*LyzM*−/−^ mice and control mice by RT-PCR. (Supplemental Fig. 1A). To confirm loss of IRS2 protein expression, we performed immunofluorescence staining in control BMDM and those derived from *Irs2*^*LyzM*−/−^ mice (*Irs2*^*−/−*^ BMDMs). This showed cytoplasmic staining for IRS2 in control BMDM and a complete absence of IRS2 in *Irs2*^*−/−*^ BMDMs (Supplemental Fig. 1B).

Next, we studied the body weight and metabolic phenotypes of *Irs2*^*LyzM*−/−^ mice on a normal chow diet. At 23 weeks of age we saw no significant difference in body weight between control and *Irs2*^*LyzM*−/−^ mice of either sex (Supplemental Fig. 1C). Assessment of percentage fat mass vs body weight by EchoMRI at earlier ages showed no differences between control and *Irs2*^*LyzM*−/−^ mice (control: 10.5 ± 1.3% vs. 12.7 ± 1.4% in *Irs2*^*LyzM*−/−^ mice, n = 8, P = 0.267). However, when energy expenditure was assessed by indirect calorimetry, 8-week old male *Irs2*^*LyzM*−/−^ mice displayed significantly increased EE in both light and dark cycles (Figure 1A). Mice at this age showed a compensatory increase in food intake on a chow diet (control: 0.199 ± 0.007 g/g/day vs. 0.229 ± 0.008 g/g/day in *Irs2*^*LyzM*−/−^ mice, n = 10, P = 0.011) similar to that seen in other mouse models with increased EE such as cold exposed mice [54] or mice with genetic interventions elevating EE (e.g. [55]). By 23 weeks of age male *Irs2*^*LyzM*−/−^ mice showed a significant reduction in fat mass (Figure 1B) with a concomitant increase in lean body mass (Figure 1C) We next assessed glucose homeostasis in chow fed-*Irs2*^*LyzM*−/−^ mice. At 8–10 weeks of age *Irs2*^*LyzM*−/−^ mice had a significant reduction in both fasting glucose and insulin levels (Figure 1D, E). This was accompanied by a mild improvement in glucose clearance and insulin sensitivity (Figure 1F and ITT AUC – control: 6611 ± 311 vs. 5618 ± 225 in *Irs2*^*LyzM*−/−^ mice, n = 8, P = 0.043). Taken together, these data suggest that deletion of *Irs2* in myeloid cells leads to a mild beneficial metabolic phenotype on a chow diet associated with increased energy expenditure.

**Figure 1:**
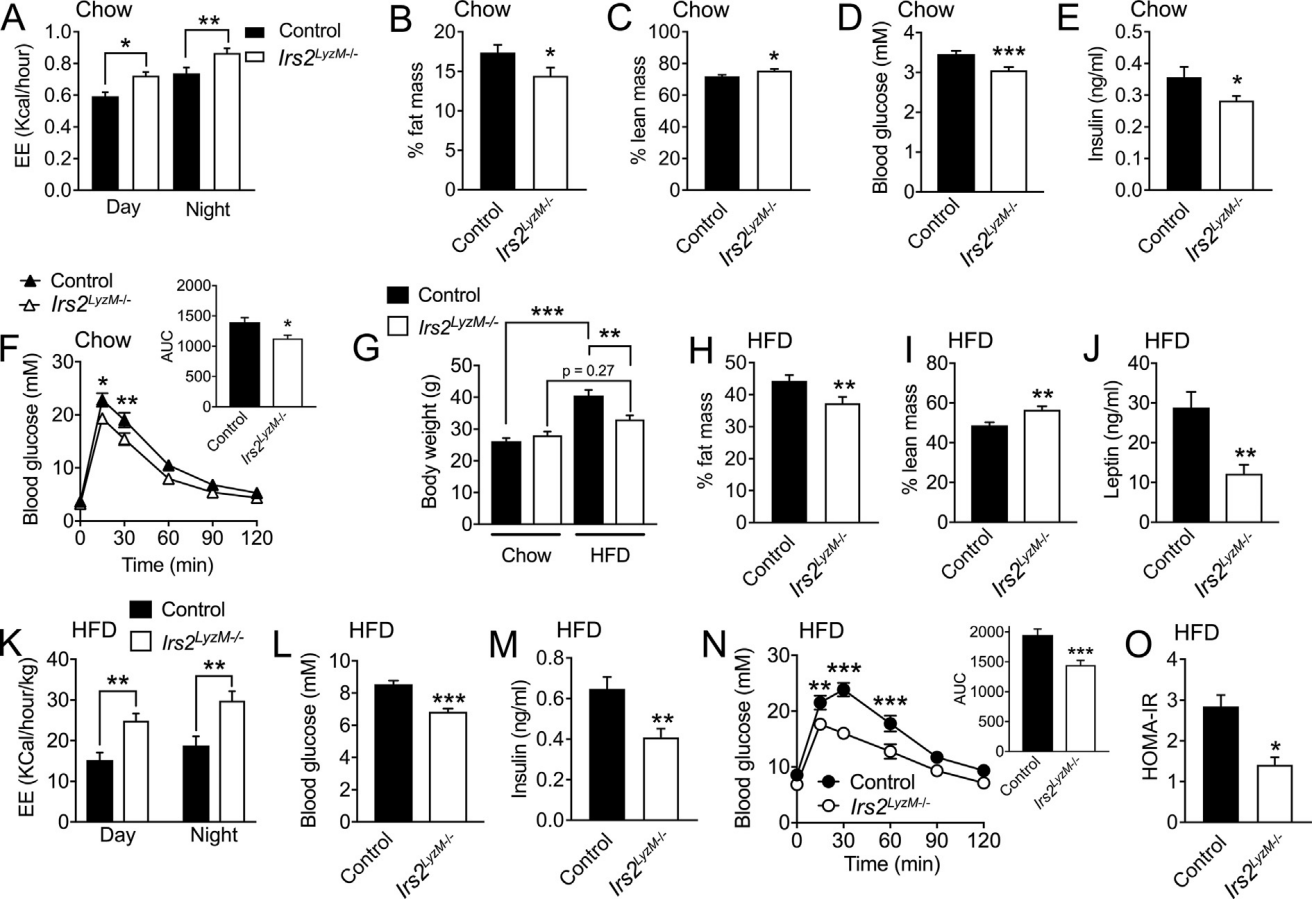
**Physiological parameters in *Irs2***^***LyzM*−/−**^**mice fed chow or HFD.** (**A**) Energy expenditure (EE) in 8-week old control and *Irs2*^*LyzM*−/−^ males during both the day and night cycles (n = 22–26). (**B, C**) Percentage fat mass (**B**) and percentage lean mass (**C**) in 23 weeks old control and *Irs2*^*LyzM*−/−^ male mice on chow diet (n = 23–24). (**D**) Blood glucose levels in 8-week old control and *Irs2*^*LyzM*−/−^ female mice following an overnight fast (n = 20). (**E**) Serum insulin levels from 8-week old control and *Irs2*^*LyzM*−/−^ female mice following an overnight fast (n = 12–20). (**F**) Glucose tolerance in overnight fasted 8-week old female control and *Irs2*^*LyzM*−/−^ mice (n = 8–9). Inset shows means of area under the curve (AUC) for both genotypes. (**G**) Body weight of control and *Irs2*^*LyzM*−/-^ females fed either chow or high fat diet (HFD) from 8 weeks of age for 15 weeks (n = 8–11 for chow and n = 23 for HFD). (**H, I**) Percentage fat mass (**H**) and percentage lean mass (**I**) in 23-week old control and *Irs2*^*LyzM*−/−^ female mice fed HFD for 15 weeks (n = 23). (**J**) Serum leptin levels following an overnight fast in control and *Irs2*^*LyzM*−/−^ female fed HFD for 15 weeks (n = 10–12). (**K**) Energy expenditure (EE) values normalized to body weight in 23-week old control and *Irs2*^*LyzM*−/−^ females fed HFD for 15 weeks (n = 18–20). (**L, M**) Blood glucose (L) and serum insulin levels (M) in overnight fasted 16-week old control and *Irs2*^*LyzM*−/−^ females fed HFD for 8 weeks (n = 20–23). (**N**) Glucose tolerance in 6-hour fasted 16-week old control and *Irs2*^*LyzM*−/−^ female mice fed HFD for 8 weeks (n = 21). Inset shows means of area under the curve (AUC) for both genotypes. (**O**) Homeostatic model assessment for insulin resistance (HOMA-IR) calculated from fasted blood glucose and serum insulin levels from mice in L and M (n = 20). In all panels, data presented are mean ± SEM with Student's t test for all panels except F, G and O, where ANOVA with repeated measure was used. (*P < 0.05, **P < 0.01 and ***P < 0.001 relative to control mice).

To assess the impact of these phenotypic changes under conditions of over-nutrition, we studied 8-week-old mice exposed to high fat diet (HFD) for 15 weeks. Control animals gained significantly more body weight on HFD compared to chow but in contrast *Irs2*^*LyzM*−/−^ mice displayed partial resistance to weight gain (Figure 1G). Consistent with this observation *Irs2*^*LyzM*−/−^ mice had reduced percentage fat mass, increased percentage lean mass and, as an indirect measure of body fat, reduced leptin levels (Figure 1H–J). *Irs2*^*LyzM*−/−^ mice also displayed increased energy expenditure on HFD, when corrected for differences in body weight (Figure 1K). These increases in energy expenditure in *Irs2*^*LyzM*−/−^ mice were also statistically significant in both the day (P = 0.007) and night (P = 0.003) cycles when using GLM (see methods 2.9) to demonstrate that genotype is a statistically significant independent contributing factor to the observed difference in EE, even after appropriately correcting for the effect of bodyweight. Assessment of glucose homeostasis in HFD-fed *Irs2*^*LyzM*−/−^ mice showed that these animals had lower fasting glucose and insulin levels and improved glucose clearance (Figure 1L–N). Insulin sensitivity as indicated by ITT (ITT AUC on HFD – control: 10451 ± 616, n = 15 vs. 8391 ± 615, n = 8 in *Irs2*^*LyzM*−/−^ mice, P = 0.044) and HOMA-IR was also improved compared to control mice (Figure 1O). Together these studies show that mice lacking *Irs2* in myeloid cells have partial resistance to both HFD-induced weight gain and the associated disruption of glucose homeostasis.

### 3.2 Loss of *Irs2* in myeloid cells is associated with an anti-inflammatory macrophage phenotype but does not alter macrophage numbers in WAT

**Figure 2:**
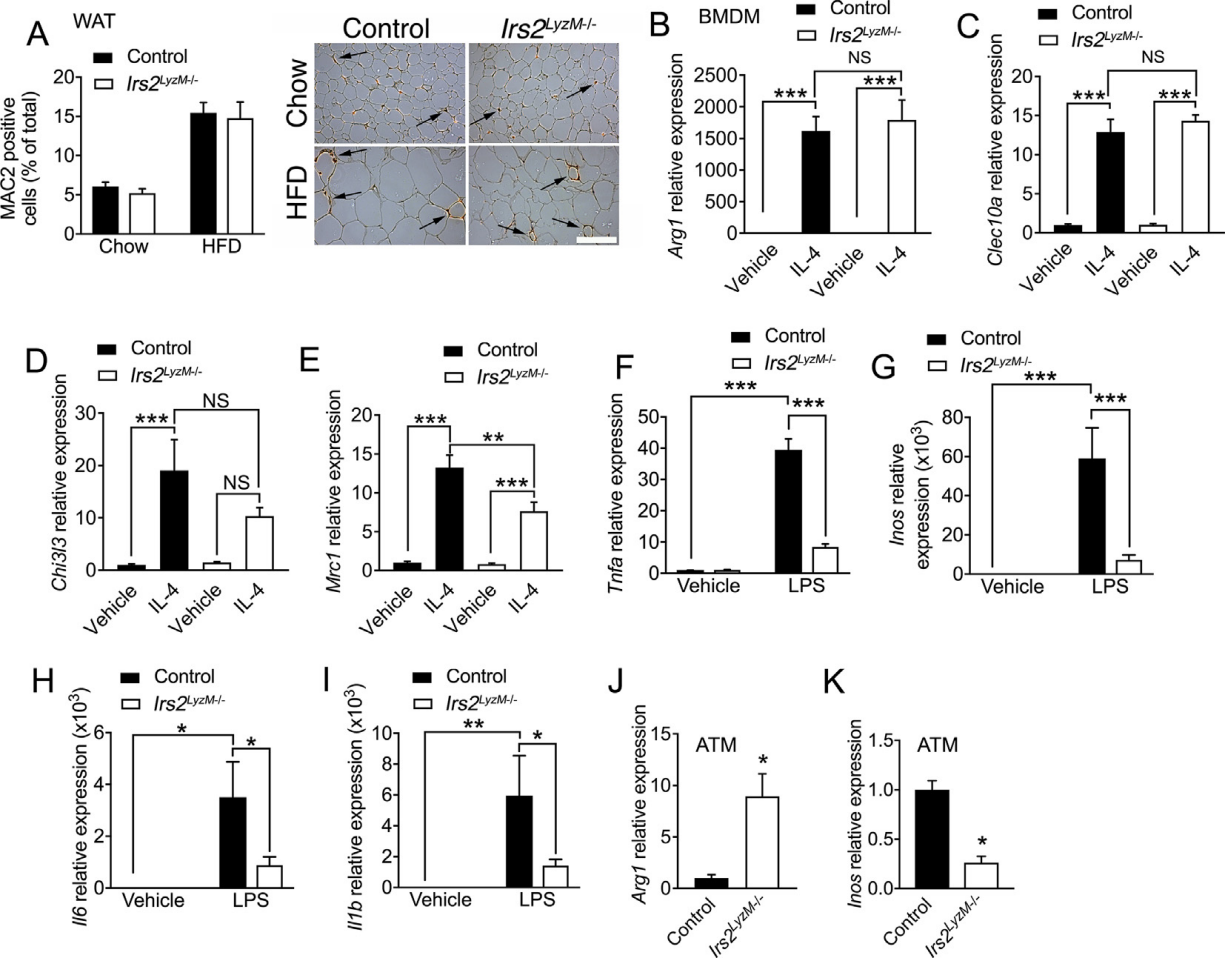
**Characterization of bone marrow derived macrophages (BMDM) from *Irs2***^***LyzM*−/−**^**mice.** (**A**) Quantification of MAC2 positive cells as percentage of total cell number in perigonadal white adipose tissue of control and *Irs2*^*LyzM*−/−^ mice on chow (n = 8–9) or high fat diet (HFD) (n = 5). Representative images are shown in the right-hand panel, with arrows indicating the presence of macrophage crown-like structures visualized by MAC2 immunohistochemical staining. Scale bar = 500 μm. (**B–E**) Quantitative RT-PCR analysis of mRNA levels of M2 markers in control and *Irs2*^*LyzM*−/−^ BMDM treated with vehicle or interleukin 4 (IL4, 10 ng/ml, 24 h). *Hprt* mRNA expression was used as an internal control and data are represented relative to control/vehicle (n = 4–7). (**F–I**) Analysis of mRNA levels of M1 inflammatory markers with LPS treatment (100 ng/ml, 24 h) (n = 4–6). (**J, K**) *Arg1* and *Inos* mRNA levels in control and *Irs2*^*LyzM*−/−^ adipose tissue macrophages (ATM) isolated from HFD-fed mice. Data are average of two independent RT-PCR experiments and ATMs were collected from a pool of 3–6 mice on HFD in each experiment (n = 6–12 mice per genotype total). In all panels, data presented are mean ± SEM with statistical analysis by ANOVA (A–I) or Student's t test (J, K). (*P < 0.05, **P < 0.01 and ***P < 0.001, relative to control BMDM/control BMDM with vehicle treatment).

The beneficial metabolic effects seen in *Irs2*^*LyzM*−/−^ mice on HFD prompted us to assess whether alterations in macrophage numbers in WAT might underlie these changes as previous studies have shown that myeloid deletion of the insulin receptor leads to lowered inflammation both in adipose tissue and systemically by reducing macrophage recruitment into WAT [31]. However, on both chow and HFD, WAT macrophage numbers were equivalent in control and *Irs2*^*LyzM*−/−^ mice (Figure 2A). Polarization of macrophages away from a pro-inflammatory CAM/M1 phenotype towards an anti-inflammatory AAM/M2 phenotype also reduces adipose tissue dysfunction [56]. Therefore, we next examined the mRNA expression of candidate markers of M1/M2 polarization in BMDM isolated from control and *Irs2*^*LyzM*−/−^ mice in response to IL4 or LPS stimulation. In response to IL4 we found equivalent expression of *Arg1* and *Clec10a* in *Irs2*-deficient BMDM and mild attenuation of the induction of *Chi3l3* and *Mrc1* (Figure 2B–E). In contrast to the limited M2 changes we detected significant reductions in the expression of M1 markers *Tnfa*, *Inos*, *Il6*, and *Il1b* in response to LPS in *Irs2*-deficient macrophages, suggesting a reduction in the pro-inflammatory phenotype in these cells (Figure 2F–I). When we examined gene expression in adipose tissue macrophages (ATM) from HFD fed mice we found increased *Arg1* expression and down-regulated *Inos* expression in *Irs2*^*LyzM*−/−^ mice (Figure 2J, K). Taken together, these findings suggest that deletion of *Irs2* in macrophages results in a markedly reduced pro-inflammatory response to LPS in macrophages rather than a broadly enhanced M2 profile when assessed by RT-PCR. Furthermore, there were no alterations in macrophage numbers in WAT upon deletion of *Irs2*.

### 3.3 Loss of *Irs2* in myeloid cells is associated with increased browning in BAT and WAT

**Figure 3:**
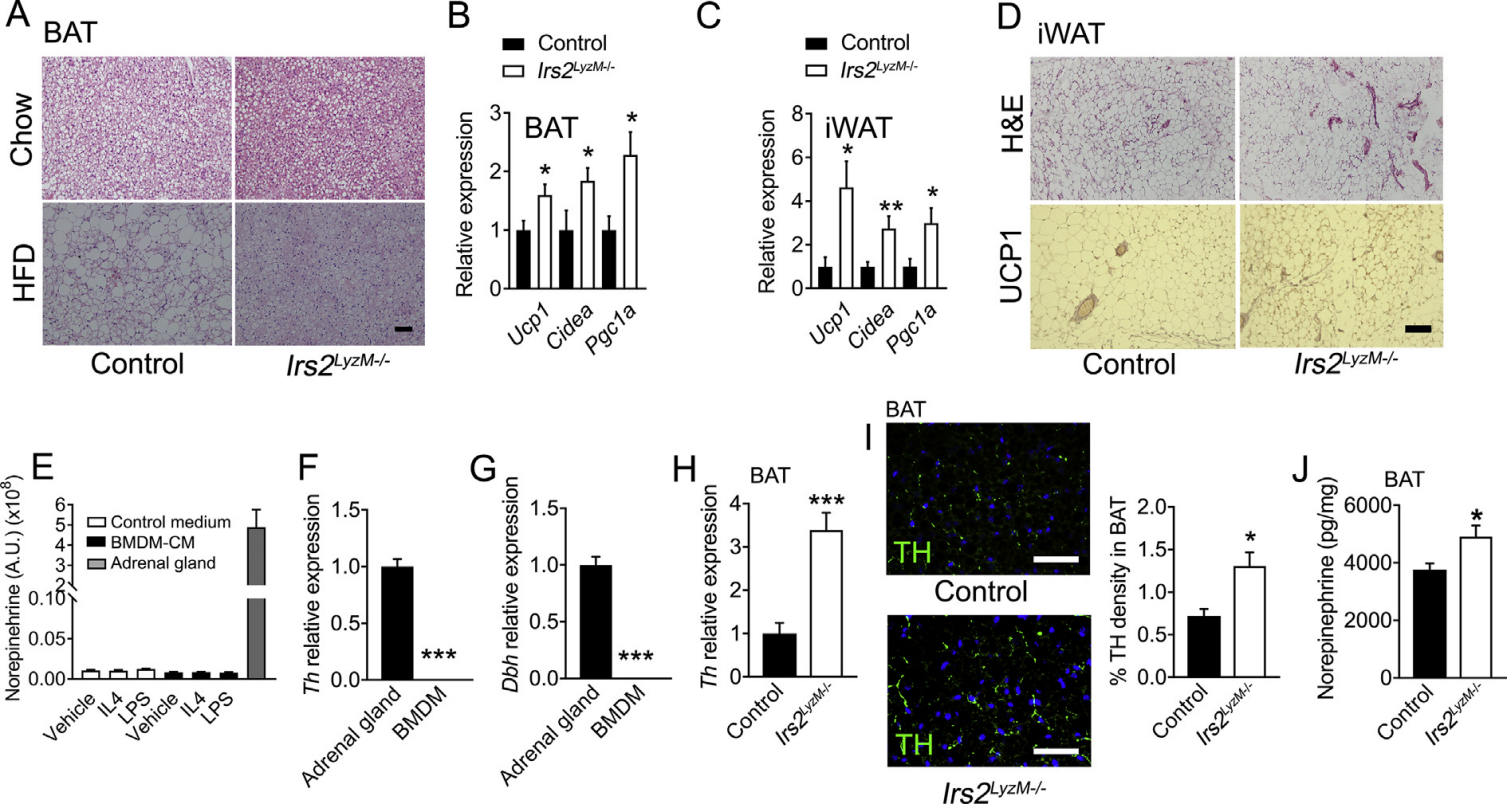
**Assessment of brown adipose tissue function in *Irs2***^***LyzM*−/−**^**mice.** (**A**) Representative hematoxylin and eosin (H&E) staining of interscapular brown adipose tissue (BAT) from control and *Irs2*^*LyzM*−/−^ mice fed either chow (top panel) or high fat diet (HFD) (lower panel) (n = 4 per group). Scale bar = 500 μm. (**B**) Quantitative RT-PCR analysis of mRNA levels of BAT genes in *Irs2*^*LyzM*−/−^ relative to control mice on chow diet. *Gapdh* mRNA expression was used as an internal control (n = 6–8). (**C**) Analysis of mRNA levels as in B of brown adipose genes in inguinal WAT (iWAT) of *Irs2*^*LyzM*−/−^ relative to control mice on chow diet. *Gapdh* mRNA expression was used as an internal control (n = 9–11). (**D**) Representative H&E staining (top panel) and immunostaining of UCP1 (lower panel) in iWAT of control and *Irs2*^*LyzM*−/−^ chow-fed mice (n = 4). Scale bar = 500 μm. (**E**) Norepinephrine levels in non-conditioned medium (Control medium) and BMDM-conditioned medium (BMDM-CM) with vehicle, IL4 and LPS treatment. Lysates of mouse adrenal gland were used as a positive control (n = 4 per group). (**F, G**) Quantitative RT-PCR analysis of *Th* (F) and *Dbh* (G) mRNA levels measured in BMDM relative to adrenal gland, both from control mice. *Hprt* mRNA expression was used as an internal control (n = 7). (**H**) Tyrosine hydroxylase (*Th*) mRNA levels in BAT from *Irs2*^*LyzM*−/−^ relative to control mice. *Gapdh* mRNA expression was used as an internal control (n = 6–8). (**I, images**) TH immunostaining (green) in BAT sections from control and *Irs2*^*LyzM*−/−^ mice. DAPI was used to visualize nuclei (blue). Scale bar = 50 μm. (**I, bar chart**) Quantification of percentage TH density in BAT sections (n = 3–4 mice/group and 6–12 fields of view per mouse). (**J**) Norepinephrine levels normalized to total protein content in BAT from control and *Irs2*^*LyzM*−/−^ mice (n = 16). In all panels, data presented are mean ± SEM with statistical analysis by Student's t test. (*P < 0.05, **P < 0.01 and ***P < 0.001 relative to control mice).

We next undertook studies to investigate the mechanisms by which loss of IRS2 signaling in myeloid cells might cause increased in energy expenditure in *Irs2*^*LyzM*−/−^ mice. BAT thermogenesis has been suggested to be regulated by macrophage polarization status with M2 macrophages reported to release catecholamines in an IL4-dependent manner [17] although these studies have been recently challenged [18]. We found reduced lipid storage as assessed morphologically in BAT in *Irs2*^*LyzM*−/−^ mice on both chow and HFD suggesting that there was increased activity of BAT metabolism (Figure 3A). Consistent with this histological finding, we found increased mRNA expression of *Ucp1* and other markers of BAT activation including *Cidea* and *Pgc1a* in *Irs2*^*LyzM*−/−^ mice on chow diet (Figure 3B). We also examined iWAT for indications of browning in this fat depot and found increased expression of *Ucp1*, *Cidea* and *Pgc1a* (Figure 3C). UCP1 protein levels as assessed by immunostaining were also increased in iWAT of *Irs2*^*LyzM*−/−^ mice (Figure 3D). Taken together, these observations indicate that *Irs2*^*LyzM*−/−^ mice had upregulated BAT function and browning of WAT.

Catecholamines play a central role in the regulation of BAT activity and WAT browning. Therefore, we next investigated whether macrophages per se might be a source of catecholamines underlying the increased browning in BAT and WAT in *Irs2*^*LyzM*−/−^ mice. Initially, we used ELISAs to measure epinephrine and norepinephrine in conditioned medium from control and *Irs2* null BMDM treated with either IL4 or LPS, but we were unable to reliably detect these catecholamines with these assays under these experimental conditions (data not shown). Therefore, we used a liquid chromatography mass spectroscopy approach to determine whether BMDM produced catecholamines. However, we were unable to detect these species at levels above those seen in media alone under the same experimental conditions despite being readily able to detect them in adrenal gland extracts (Figure 3E). To further investigate these findings, we examined the expression of the catecholamine biosynthesis enzymes *Th* and *Dbh* expression in BMDM from control mice using RT-PCR. We validated that our primers were functional using adrenal glands as a control but were unable to detect any expression of catecholamine biosynthetic enzymes in macrophages (Figure 3F, G). Taken together, these findings suggest that macrophages are not a source of norepinephrine that might underlie the upregulated BAT function and browning of WAT seen in *Irs2*^*LyzM*−/−^ mice. Furthermore, our findings are consistent with some recent observations showing that macrophage populations do not express *Th* or produce catecholamines [18], [20].

### 3.4 Adipose tissue macrophages associate with sympathetic nerves: *Irs2*^*LyzM*−/−^ mice display increased density of TH-positive neurons and elevated norepinephrine levels in BAT

Recent findings have suggested that CX3CR1-expressing macrophages in BAT may associate with TH-positive axons and that macrophage-specific deletion of the transcriptional repressor *Mecp2*, impairs BAT sympathetic innervation [45]. Furthermore, separate studies have shown both ATM and a novel macrophage sub-population (termed sympathetic-associated macrophages: SAMs) directly interact with WAT sympathetic nerves and in part regulate adipose tissue lipolysis by controling local catecholamine scavenging and catabolism [20]. In our own studies, we confirmed that CX3CR1-expressing macrophages abutted BAT TH-positive neurons (Supplemental Fig. 1D). Furthermore, we found increased *Th* mRNA expression and TH-nerve fibre density in the BAT of *Irs2*^*LyzM*−/−^ mice suggesting an increase in sympathetic innervation of BAT (Figure 3H, I). Consistent with these observations, BAT norepinephrine levels were elevated in *Irs2*^*LyzM*−/−^ mice (Figure 3J). Together these findings suggest that macrophages lacking *Irs2* are able to positively influence WAT/BAT sympathetic nerve function leading to enhanced local release of norepinephrine and increase of thermogenic capacity in adipose tissues.

### 3.5 RNA sequencing analysis of *Irs2*-deficient macrophages reveals gene expression signatures of an anti-inflammatory phenotype and increased sympathetic activity in *Irs2*^*LyzM*−/−^ mice

**Figure 4:**
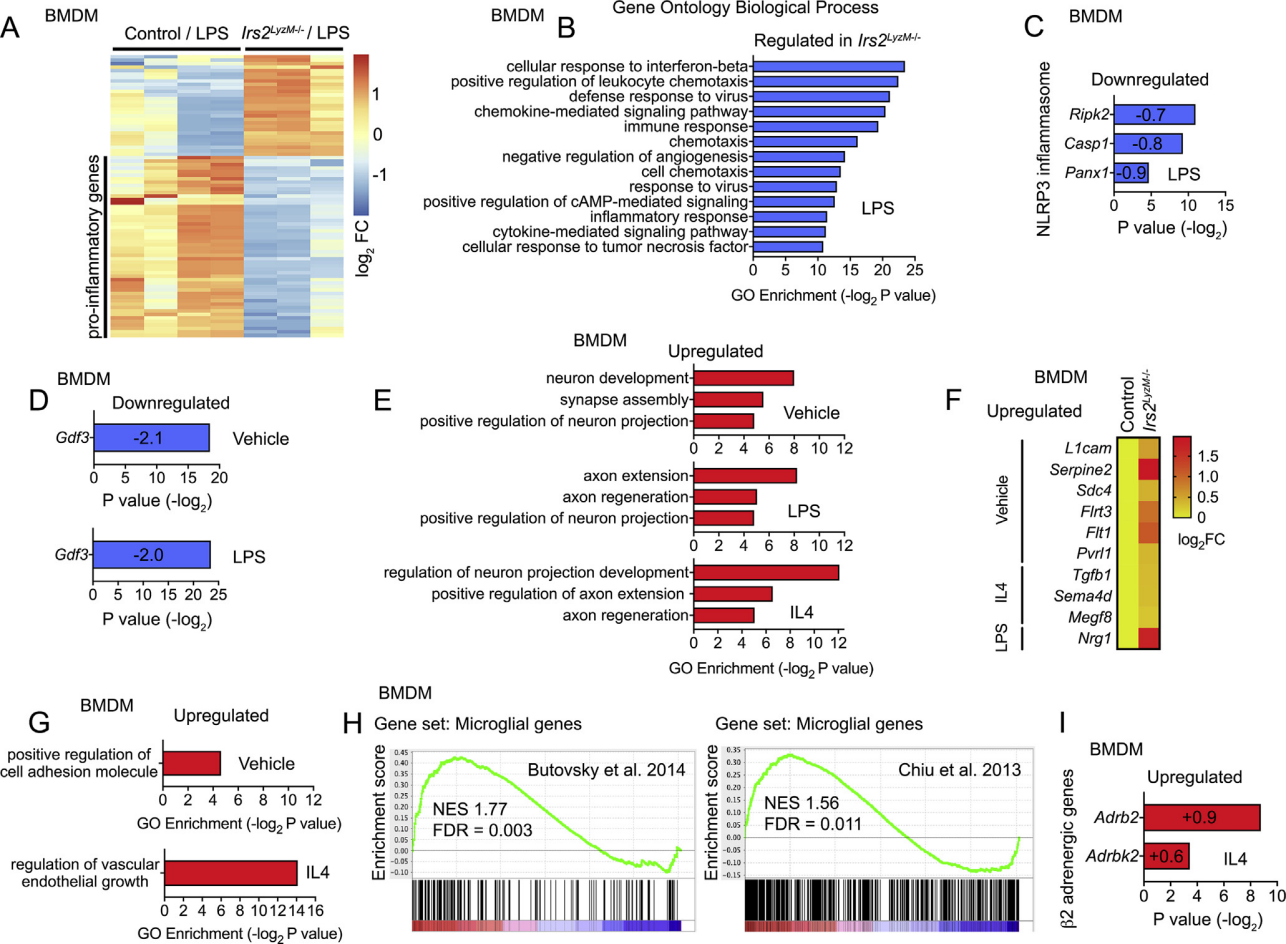
**mRNA sequencing analysis of control and *Irs2***^***LyzM*−/−**^**BMDM.** (**A**) Heat map showing differentially regulated genes in *Irs2*^*LyzM*−/−^ vs control BMDM following 24 h LPS treatment from RNA-seq analysis, including a large subset of downregulated pro-inflammatory genes in the *Irs2*^*LyzM*−/−^/LPS group (n = 4 for control, 3 for *Irs2*^*LyzM*−/−^). (**B**) Gene ontology (GO) categories of top 200 significantly regulated genes in *Irs2*^*LyzM*−/−^ BMDM following 24 h treatment with LPS. (**C**) Differential expression of NLRP3 inflammasome-associated genes in LPS-treated *Irs2*^*LyzM*−/−^ vs LPS-treated control BMDM. Log_2_ fold change for each gene is shown in its respective bar. (**D**) Differential expression of *Gdf3* in both vehicle and LPS-treated *Irs2*^*LyzM*−/−^ relative to control BMDM with respective treatments. Log_2_ fold change is shown in each respective bar. (**E**) GO categories with significant enrichment related to nerve growth and function in *Irs2*^*LyzM*−/−^ relative to control BMDM with either vehicle, LPS or IL4 (overnight) treated conditions. (**F**) Heat map showing upregulated neuronal-associated genes (secreted and cell surface proteins involved in cell adhesion and cell-to-cell interactions) in *Irs2*^*LyzM*−/−^ vs control BMDM under various conditions. (**G**) GO categories with significant enrichment in *Irs2*^*LyzM*−/−^ relative to control BMDM under various conditions. (**H**) Gene set enrichment analysis showing increase of microglial markers in IL4-treated *Irs2*^*LyzM*−/−^ relative to IL4-treated control BMDM. (**I**) Differential expression of β2 adrenergic genes *Adrb2* and *Adrbk2* in IL4-treated *Irs2*^*LyzM*−/−^ relative to IL4-treated control BMDM. Log_2_ fold change for each gene is shown in its respective bar. In panels B–E and also G and I, P values for GO enrichment categories or individual genes are shown, calculated as described in the methods (section 2.8).

To try to gain broader insights into how deletion of *Irs2* in macrophages might impart a beneficial metabolic phenotype and influence BAT function, we undertook RNA sequencing studies in BMDM from control and *Irs2*^*LyzM*−/−^ mice. We focused our analysis on 1) macrophage inflammatory processes that may influence the ability of macrophages to scavenge and catabolize catecholamines as well as more broadly influence adipose tissue function and 2) processes by which macrophages may influence sympathetic innervation of BAT. In BMDMs treated with LPS, deletion of *Irs2* was associated with a significantly reduced expression of pro-inflammatory gene categories as shown by gene ontology (GO) analysis. Down-regulated GO categories included regulation of cellular response to tumor necrosis factor, cytokine-mediated signaling pathway, inflammatory response, and cellular response to interferon beta (Figure 4A, B). Significantly down-regulated genes belonging to these categories included *Inos*, the *Nfkb*-related gene *Nfkbib*, *Tnfa*, IL-related genes (*Il1a*, *Il19*, *Il7r*, *Il15*, *Il10*, *Il1rn*, *Il7r*, and *Il27*), the *Tgf* beta receptor type I and a range of cytokines and chemokines (*Cxcl9, Cxcl10, Ccl2, Ccl4, Ccl5, Ccl7, Clc8, Ccrl2*, *and Ccl12* (Figure 4A, B, and Supplemental Table 2)). The expression of genes associated with the NLRP3 inflammasome *Ripk2*, *Casp1*, and *Panx1* was also reduced (Figure 4C). These data indicate that deletion of *Irs2* in macrophages engenders a broad anti-inflammatory profile in these cells under the pro-inflammatory stress conditions of LPS treatment.

Recently it has been found that macrophages from aged mice display an inflammatory phenotype and an associated increased ability to lower WAT catecholamine levels due to a GDF3-dependent up-regulation of the catecholamine degradation machinery [19]. Consistent with these findings, relative *Gdf3* expression was significantly reduced in both untreated and LPS-treated *Irs2*^*LyzM*−/−^ BMDM compared to control BMDM. (Figure 4D). However, when we examined expression of the machinery for bioamine uptake, we did not detect *Slc6a2*, the noradrenaline transporter (Supplemental Table 2), which is consistent with recent findings [20] and suggests this is exclusively expressed in SAMs. In addition, we examined the expression of a number of genes involved in bioamine metabolism such as *Maoa, Comt*, and members of the *Aldh* and *Akr* families of enzymes involved in biogenic amine degradation. Although *Maoa* expression was reduced in LPS-treated *Irs2*^*LyzM*−/−^ BMDM, in general there was no concerted change in the expression pattern of these genes that would explain the increase in WAT catecholamine levels via effects upon macrophage-clearance of catecholamines (Supplemental Table 2).

Deletion of *Mecp2*, a nuclear transcriptional regulator implicated in Rett syndrome, in the BAT-resident CX3CR1-expressing macrophage population recently has been shown to reduce BAT sympathetic innervation [45]. In contrast, we demonstrate increased TH-positive nerve fibre density in this tissue in *Irs2*^*LyzM*−/−^ mice. Together these findings indicate that intrinsic macrophage signaling events might regulate pathways directly involved in nerve growth and maintenance. Consistent with this idea, in untreated *Irs2*^*LyzM*−/−^ BMDM we found increases in the expression of genes in GO categories including positive regulation of neuron development, synapse assembly and neuron projection (Figure 4E). In *Irs2*^*LyzM*−/−^ BMDM treated with IL4 we found increased expression in GO categories including regulation of neuron projection development and positive regulation of axon extension and axon regeneration (Figure 4E). In LPS-treated *Irs2*^*LyzM*−/−^ BMDM, we found increased expression in GO categories including positive regulation of neuron projection and axon extension and remodeling (Figure 4E). While these categories included genes classically expressed in neurons they also included genes for secreted proteins and cell surface proteins involved in cell adhesion and cell-to-cell interaction processes through which macrophages could potentially directly be involved in the regulation of neuronal function. The genes in the GO categories involved included: *L1cam*, a cell adhesion molecule involved in neurite outgrowth [57]; *Serpine2*, which promotes neurite extension [58]; *Sdc4*, which plays a role in neuron adhesion processes [59]; *Flrt3*, which is involved in axon guidance [60]; *Flt1*, which has neurotrophic effects [61]; *Pvrl1*, an adhesion molecule with roles in glia–neuron interactions [62]; *Tgfb1*, which is involved in a number of elements of neuronal function [63]; *Sema4d*, a semaphorin that acts a soluble neurotrophic factor [64]; *Megf8*, a transmembrane EGF-like protein implicated in neural growth [65] and *Nrg1*, a nerve growth factor [66] (Figure 4F). Gene expression categories related to cell adhesion and to vascular development, which may also be involved in the control of tissue innervation [67], were also upregulated in *Irs2*^*LyzM*−/−^ BMDM (Figure 4G). Together these findings suggest that deletion of *Irs2* in macrophages potentially creates an environment in BAT that enhances sympathetic innervation.

Interestingly, we also found an increase in the expression of microglial markers in IL4 treated *Irs2*^*LyzM*−/−^ BMDM including *Cx3cr1* (Figure 4H [68], [69]), suggesting that there could be an increased association of these cells with nerves although we did not detect increased macrophage numbers in adipose tissue. Furthermore, we note that *Irs2* is enriched in SAMs compared to other macrophage populations [20] again suggesting a role for this signaling molecule in these cells. We also found a significant increase in the expression of the β2 adrenergic receptor (*Adrb2*) and a non-significant increase in the β adrenergic receptor kinase 2 (*Adrbk2*) in IL4-treated *Irs2*^*LyzM*−/−^ BMDM (Figure 4I). Together these expression profiles suggest that macrophages may play a positive role in tissue nerve function and that deletion of *Irs2* increases the expression of such pathways indicating potential bidirectional interplay between macrophages and sympathetic nerves.

### 3.6 *Irs2* expression in BMDM is increased by palmitate and deletion of *Irs2* in macrophages attenuates insulin signaling but paradoxically enhances IL4 signaling

Next, we further explored potential mechanisms underlying the above gene expression profiles showing reduced inflammatory response to LPS but a paradoxically preserved response to IL4 in *Irs2*^*LyzM*−/−^ BMDM. Insulin signaling in macrophages has been suggested to be pro-inflammatory indicating that *Irs2* might mediate such events and that macrophage IRS2 may be involved in the detrimental effects of inflammatory macrophages upon insulin action in other tissues. Palmitate treatment induces an inflammatory state in BMDM and conditioned medium from these cells induces insulin resistance in muscle cells [70]. Consistent with this idea incubation of BMDM was associated with an increase in *Irs2* expression (Supplemental Fig. 1E). Recent studies in BMDM from mice with global deletion of *Irs2* have demonstrated enhanced IL4-dependent M2 marker expression, thought to be due to increased signaling via IRS1 [29]. As described above, we also found preservation and in some cases enhancement of IL4-stimulated gene expression in *Irs2*^*LyzM*−/−^ BMDM. However, we found minimal expression of *Irs1* and *Irs3* and no *Irs4* mRNA in wild type BMDM consistent with previous findings that *Irs2* is the predominant *Irs* gene in hematopoetic cells [24–26 and Supplemental Table 2], and we saw no change in expression of *Irs1* or *Irs3* mRNA upon *Irs2* deletion in our RNAseq data (*Irs1*: 0.12 log_2_FC; P = 0.91 and *Irs3*: 0.11 log_2_FC; P = 0.89). Indeed, we could not detect IRS1 protein expression in these cells by western blotting (Supplemental Fig. 1F). However, in *Irs2*^*LyzM*−/−^ BMDM, we found increased AKT phosphorylation in response to IL4 treatment (Supplemental Fig. 1G). This was consistent with recent studies [29] despite attenuation of insulin-stimulated AKT phosphorylation (Supplemental Fig. 1H), indicating that normally IRS2 signaling in part plays a role in restraining IL4 signaling or that loss of IRS2 allows an alternative signaling pathway to be engaged that mediates effects upon gene expression driven by IL4.

## 4 Discussion

There is increasing recognition that the interplay between the immune and nervous systems plays a key role in the regulation of adipose tissue function [71]. In the current studies, we demonstrate that deletion in *LyzM*-expressing macrophage populations of *Irs2*, a major mediator of IL4 and insulin signaling, leads to resistance to diet-induced obesity via increased BAT thermogenesis through increased sympathetic innervation and increased local catecholamine availability. Underpinning these observations, we find that *Irs2*-deficient BMDM do not express catecholamine biosynthetic machinery but, when challenged with an inflammatory stimulus, display a transcriptional profile consistent with reduced inflammation together with alterations in the expression of genes that are involved in the regulation of tissue innervation, sympathetic nerve function, and adipose tissue lipolysis. Our studies therefore links insulin and IL4 signaling through IRS2 in macrophages to the regulation of systemic metabolism via control of adipose tissue sympathetic nerve function, adding new insights to the signaling processes involved in this neuro–immune interaction.

Previously, a series of studies has suggested that AAM produce catecholamines in an IL4-dependent manner to regulate BAT and WAT thermogenic programs and adipocyte metabolism [17]. In this work, mice with myeloid-restricted deletion of *Il4ra* displayed impaired M2 polarization and defective cold adaptive thermogenesis associated with a reduced production of catecholamines by macrophages [17]. However, recent evidence has challenged this model with a failure to detect either catecholamine biosynthetic apparatus in adipose tissue macrophages or beneficial effects of chronic IL4 treatment upon energy expenditure [18], [20]. While we found both increased BAT browning and an associated increase in adipose tissue noradrenaline levels in our mutant mice, we did not detect the machinery for catecholamine synthesis in BMDM or noradrenaline in BMDM conditioned medium at levels above that seen in medium alone. Recent studies have also suggested that macrophages per se are not the source of catecholamines in adipose tissue [18], [20], [72], although another recent paper has demonstrated *Th* expression in ATM [73]. Although our studies are consistent with the idea that macrophages in adipose tissue may not produce catecholamines, as we have performed many studies on BMDM rather than ATM our work is not definitive about this issue as resident macrophages in tissues may display distinct phenotypes from BMDM [74]. Future work will be required to address this specific question in *Irs2*^*LyzM*−/−^mice.

Our studies do suggest, however, that alterations in insulin and IL4 signaling may impact upon macrophage biology to engender changes in adipose tissue function via sympathetic innervation. These observations are complicated by our findings that deletion of *Irs2* in macrophages causes polarization towards an anti-inflammatory phenotype with persistent rather than attenuated signaling downstream of IL4. This is consistent with recent findings in macrophages from mice with global deletion of *Irs2* which also displayed a paradoxical enhancement of IL4 signaling [29] although, in our case, we did not detect upregulation of *Irs1* expression in *Irs2*-deficient macrophages. It therefore remains to be seen what mediates the preserved IL4 signaling in *Irs2*-deficient macrophages in our mouse model, with other adaptor molecules downstream of IL4 receptor signaling potentially playing a role. *Irs2* loss has been shown to cause enhanced rather than attenuated functional end-points, including enhanced learning and memory [75]. This in turn leads to positive effects on physiology, suggesting that in certain circumstances IRS2 may act as a brake upon specific signaling processes [75].

Taken together, our data illustrate that altered insulin and IL4 signaling in macrophages can have beneficial metabolic effects acting via alterations in sympathetic nerve function rather than release of catecholamines by macrophages per se. Recent studies have also revealed potential alternative mechanisms by which this macrophage-mediated regulation of adipose tissue sympathetic nerve function occurs. These include the scavenging and catabolism of catecholamines or the direct control of adipose tissue sympathetic innervation [19], [20], [45]. For example, it has been demonstrated that a GDF3-dependent mechanism mediates the reduction of catecholamine-induced lipolysis that occurs with ageing [19]. This study showed that age-related NLRP3 inflammasome activation increased the ability of macrophages to scavenge catecholamines in adipose tissue with elevated expression of MAOA occurring via GDF3, thereby resulting in reduced adipose tissue lipolysis [19]. Consistent with these findings we show that *Irs2*-deficient BMDM have reduced expression of *Gdf3* which would potentially result in increased adipose tissue catecholamine availability, although it should be noted that we have not studied gene expression in ATMs per se. A parallel finding has been the identification of a specialized myeloid sub-population that is associated with sympathetic nerves [20]. These SAMS scavenge catecholamines via SLC6A2-dependent uptake and MAOA degradation pathways and abrogation of SAM function leads to increased BAT recruitment and WAT browning suggesting they may have a role in regulating adiposity [20]. While we have not isolated SAMs, we find that elements of the catecholamine scavenging machinery are expressed in BMDM (although not *Slc6a2*) although we do not detect concerted alterations of these genes upon *Irs2* deletion in these cells that might reduce macrophage uptake and clearance of catecholamines. We also find that macrophages can be associated with BAT sympathetic nerves consistent with the findings of others and demonstrating the potential for the direct regulation of sympathetic nerve function by macrophages. Interestingly, while it appears that SAMs have a pro-inflammatory phenotype [20], macrophages lacking *Irs2* have an anti-inflammatory phenotype when challenged with LPS suggesting that distinct mechanisms and myeloid populations may be involved. However, it also appears that SAMs are enriched for *Irs2*-expression and express *LyzM* [20] and so it is possible that altered SAM function contributes to the phenotype seen in our mice.

While we appear to only have partial evidence for a mechanism that might involve alterations in catecholamine scavenging by macrophages, we find increased density of sympathetic nerves in BAT and a series of transcriptional changes in BMDM that might underpin this finding. These include up-regulation of genes not only in implicated in specific components of neuronal function such as axon guidance and development but also in processes such as vascularization and cell adhesion, which may play more generic roles in tissue innervation. That macrophages can directly interact with the processes involved in regulating BAT sympathetic nerves has been illustrated recently in mice with macrophage deletion of *Mecp2* [45]. The underlying mechanisms appear to involve semaphorin signaling cross-talk between macrophages and nerves and specifically macrophages expressing CX3CR1 [45]. We do not find directly equivalent gene expression alterations in semaphorin pathways in *Irs2*^*LyzM*−/−^ BMDM, but, interestingly, these cells were enriched for microglial markers such as *Cxc3r1* potentially, suggesting an increased propensity to interact with nerves.

While our work and that of others suggest that adipose tissue macrophages directly interact with sympathetic nerves to regulate catecholamine tone in this tissue, there are several lines of evidence that macrophages with an inflammatory profile may impair the ability of BAT to respond appropriately to thermogenic stimuli. For example, in vitro studies and analysis of macrophages from mice under HFD feeding shows that TNFA and IL1B directly suppress the expression of UCP1 in both BAT and WAT [76], [77]. Therefore, the markedly reduced inflammatory profile seen in *Irs2*^*LyzM*−/−^ BMDM could contribute to the enhanced BAT activity through direct transcriptional effects on *Ucp1* and other components of the thermogenic programme in addition to indirect effects via adipose tissue sympathetic nerve function. Upregulation of *Irs2* by palmitate, a pro-inflammatory stimulus for BMDM that leads to secretion of insulin resistance-causing factors from macrophages, further supports the idea that deletion of *Irs2* reduces macrophage inflammation and that this may be involved in the beneficial metabolic effects including improved insulin sensitivity.

It should also be noted that the *LyzM*-*Cre* mouse utilized in our studies deletes in other myeloid cell lineages. These include neutrophils which have also been implicated in the regulation of adipose tissue function and obesity-related insulin resistance [7], [8], [9], [78]. Therefore, a limitation of our study is that the in vivo phenotypes reported could potentially involve a contribution from the cell types distinct from macrophages in which *LyzM*-*Cre* is expressed.

In summary our studies indicate that the deletion of *Irs2* in macrophages has beneficial metabolic effects stemming from increased activity of BAT and browning of WAT depots at ambient temperatures due to an underlying increase in adipose tissue catecholamine function. This in turn is due to pleiotropic effects upon macrophage function including a markedly reduced inflammatory profile, the expression of genes that may be involved in catecholamine scavenging and the development of an appropriate cellular environment for enhanced adipose tissue sympathetic innervation. Consistent with recent studies we show that macrophages do not produce catecholamines per se but that alteration of a component of both IL4 and insulin signaling in the *LyzM* population of macrophages can have profound effects on systemic metabolism. Therefore, our work adds to the growing evidence that neuro–immune interactions play a central role in adipose tissue biology and the pathophysiology of obesity and insulin resistance while identifying potential new cellular and molecular events to target for therapeutic benefit in these conditions.

## Declarations of interest

None.

## Author contributions

MTR, SJM, and DJW designed the research. MTR, SJM, SMAP, AIC, ASHC, DH, JG, EEI, CS, SK, and MCW performed and analysed experiments. VKY contributed reagents and tools. MTR, SJM, CS, VKY, AVP, SV, CF, and DJW drafted and/or wrote the manuscript. MTR, VKY, CF, and DJW provided funding. DJW supervised the work.

## Figures and Tables

**Table:** 

List of probes used in RT-PCR. (TaqMan, Applied Biosystems, all *Mus Musculus)*.	
Interleukin 1 beta *(Il1b)*	Mm01336189_m1
Interleukin 6 *(Il6)*	Mm00446190_m1
Inducible nitric oxide synthase *(Inos)*	Mm00440502_m1
Tumor necrosis factor alpha *(Tnfa)*	Mm00443258_m1
Arginase 1 *(Arg1)*	Mm00475988_m1
Chitinase 3-like-3 *(Chi3l3)*	Mm04213363_m1
Mannose receptor 1 *(Mrc1)*	Mm00485148_m1
C-type lectin domain family 10 member A *(Clec10A)*	Mm00546124_m1
Hypoxanthine phosphoribosyltransferase *(Hprt)*	Mm03024075_m1
Uncoupling protein 1 *(Ucp1)*	Mm01244861_m1
Glyceraldehyde-3-phosphate dehydrogenase *(Gapdh)*	Mm99999915_g1
Peroxisome proliferator-activated receptor gamma, coactivator 1 alpha *(Pgc1a)*	Mm01208835_m1
Tyrosine hydroxylase *(Th)*	Mm00447557_m1
Cell death-inducing DNA fragmentation factor, alpha subunit-like effector A *(Cidea)*	Mm00432554_m1
Insulin receptor substrate 2 *(Irs*2)	Mm03038438_m1
Dopamine beta hydroxylase *(Dbh)*	Mm00460472_m1

**figs1:**
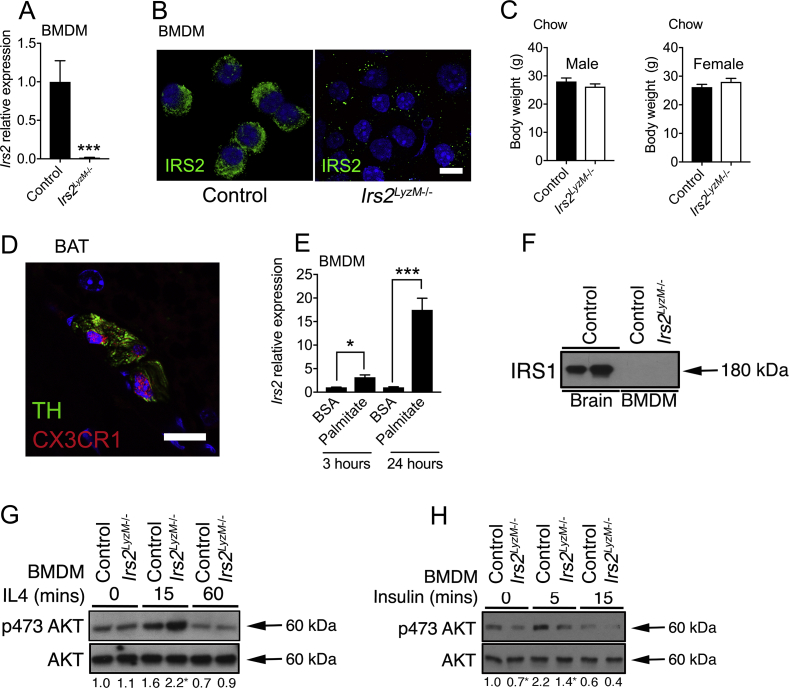
(A) Quantitative RT-PCR analysis of *Irs2* mRNA expression in control and *Irs2*^*LyzM*-/−^ BMDM. *Hprt* was used as an internal control (n = 5-6). (B) IRS2 immunostaining (green) in control and *Irs2*^*LyzM*-/−^ BMDM. DAPI was used to visualize nuclei (blue). (3 mice/genotype). Scale bar = 10 μm. (C) Body weights of control and *Irs2*^*LyzM*-/−^ male mice and female on chow diet at 23 weeks of age (n = 8). (D) TH (green) and CX3CR1 (red) immunostaining in BAT from wild type mice. DAPI was used to visualize nuclei (blue). Scale bar = 20 μm (E) Quantitative RT-PCR analysis of *Irs2* mRNA expression in control BMDM treated with palmitate for 3 or 24 hours. BSA-treated BMDM were used as a control and *Hprt* mRNA was used as an internal gene control (n = 5-6). (F) Western blotting analysis of IRS1 expression in BMDM from control and *Irs2*^*LyzM*-/−^. Brain tissue lysates were used as a positive control. (G, H) Western blotting analysis of serine 473 phosphorylation of AKT in BMDM from control and *Irs2*^*LyzM*-/−^ mice treated with IL4 (G) or insulin (H) for the times indicated. A representative blot is shown (n = 4-5). Mean values for band intensities in multiple samples quantified by densitometry are shown below the panel, relative to control samples for each time point. In all panels, data presented are mean ± SEM with Student’s t test for all panels. (*P < 0.05 and ***P < 0.001 relative to controls).
